# Early Survivorship Analysis of a Modern Anatomic Cementless Tibial Baseplate: A Multicenter Retrospective Study

**DOI:** 10.1016/j.artd.2026.102117

**Published:** 2026-08-12

**Authors:** Alexander Burbelo, Rex Lutz, Steven Fischer, Harrison Patrizio, Marcus Voigt, Liam Cleary, Trace Clark, Arjun Saxena, Alvin Ong, Matthew Bullock

**Affiliations:** a Department of Orthopaedic Surgery, Marshall University Joan C. Edwards School of Medicine, Huntington, WV; b Department of Orthopaedic Surgery, Jefferson Health New Jersey, Stratford, NJ; c Rowan University School of Osteopathic Medicine, Stratford, NJ; d Rothman Orthopaedic Institute, Philadelphia, PA

**Keywords:** Cementless, Total knee arthroplasty (TKA), Survivorship, Aseptic loosening, Porous titanium

## Abstract

**Background:**

Cementless total knee arthroplasty has seen increased adoption given the potential for durable biologic fixation. However, limited data exist on modern anatomic cementless tibial baseplate designs. This study evaluates early survivorship and functional outcomes of a novel cementless tibial baseplate compared to a cemented counterpart from the same manufacturer.

**Methods:**

A multicenter retrospective review was conducted of 394 patients undergoing primary total knee arthroplasty at 2 academic-affiliated institutions from 2018 to 2023. Of these, 183 received a porous anatomic cementless tibial baseplate (Smith & Nephew, Memphis, TN) and 211 received a Genesis II cemented tibial baseplate. Mean follow-up was 1.4 years and 2.7 years, respectively. The primary outcome was all-cause revision-free survivorship; secondary outcomes included aseptic tibial loosening and Knee Injury and Osteoarthritis Outcome for Joint Replacement scores.

**Results:**

One patient in each group required revision for aseptic tibial baseplate loosening. Two-year all-cause revision-free survivorship was 96.2% (95% confidence interval [CI]: 91.2-100) in the cementless group and 96.0% (95% CI: 92.6-99.6) in the cemented group (*P* = .92). Unadjusted Cox regression demonstrated no statistically significant difference in revision risk (hazard ratio 3.27, 95% CI 0.52–20.53; *P* = .207); however, estimates were imprecise and the hazard ratio consistently favored the cemented cohort. No significant between-group difference in Knee Injury and Osteoarthritis Outcome for Joint Replacement improvement was identified after multivariable adjustment (β = 3.46; 95% CI, −1.31 to 8.24; *P* = .154).

**Conclusions:**

This cementless tibial baseplate demonstrated low early revision and aseptic loosening rates; however, imprecise Cox regression estimates preclude conclusions regarding equivalent survivorship. Longer-term prospective studies with contemporary controls are warranted.

**Level of Evidence:**

III, Retrospective comparative cohort study.

## Introduction

The use of cementless fixation in total knee arthroplasty (TKA) has increased in recent years [1,2]. Cementless TKA offers the potential advantage of a biologic implant–bone interface, which may theoretically enhance long-term fixation [1]. Additionally, it is associated with shorter operative times while demonstrating a comparable short-term failure profile to cemented fixation [3,4]. A common cause of early cementless TKA failure is aseptic loosening, particularly involving the tibial baseplate [5]. In response, most contemporary TKA manufacturers have developed cementless tibia baseplates featuring keel and peg configurations designed to provide immediate mechanical stability and facilitate subsequent osteointegration [6,7].

Most cementless TKA systems used in the United States are produced by 4 major manufacturers [8]. One cementless TKA system designed with a keel and cruciate peg configuration has demonstrated low early aseptic loosening rates [7,9]. Conversely, a recent study involving a keel-less two-peg cementless tibial component reported an aseptic loosening rate of 2.7% [10]. In 2025, early outcomes of a modern anatomic cementless tibial baseplate system were compared to matched cemented cases, with findings suggesting higher early revision rates due to aseptic loosening and poorer patient-reported outcomes at 12 months in the cementless cohort [11].

Given the increasing interest in cementless TKA constructs, the present study aims to evaluate the early outcomes of a novel cementless tibial baseplate using multi-institutional survivorship data and to compare these results with its matched cemented counterpart. We hypothesized that this implant would demonstrate a low rate of early aseptic tibial baseplate loosening and patient-reported outcomes comparable to a modern cemented TKA system from the same manufacturer.

## Material and methods

Following institutional review board approval, a retrospective review was conducted of 394 patients who underwent TKA at 2 academic-affiliated institutions. The cohort included 183 patients who underwent TKA with a Smith & Nephew porous tibial baseplate (Memphis, TN) between January 2022 and December 2023 and 211 patients who underwent cemented TKA using a Smith & Nephew Genesis II cemented tibial baseplate (Memphis, TN) between June 2018 and January 2020. All procedures were performed by one of three fellowship-trained adult reconstruction surgeons. Inclusion criteria consisted of patients aged 18 years or older who underwent TKA using the aforementioned baseplates. Demographic variables collected included age, sex, smoking status, diabetes status, and body mass index (BMI).

**Figure 1:**
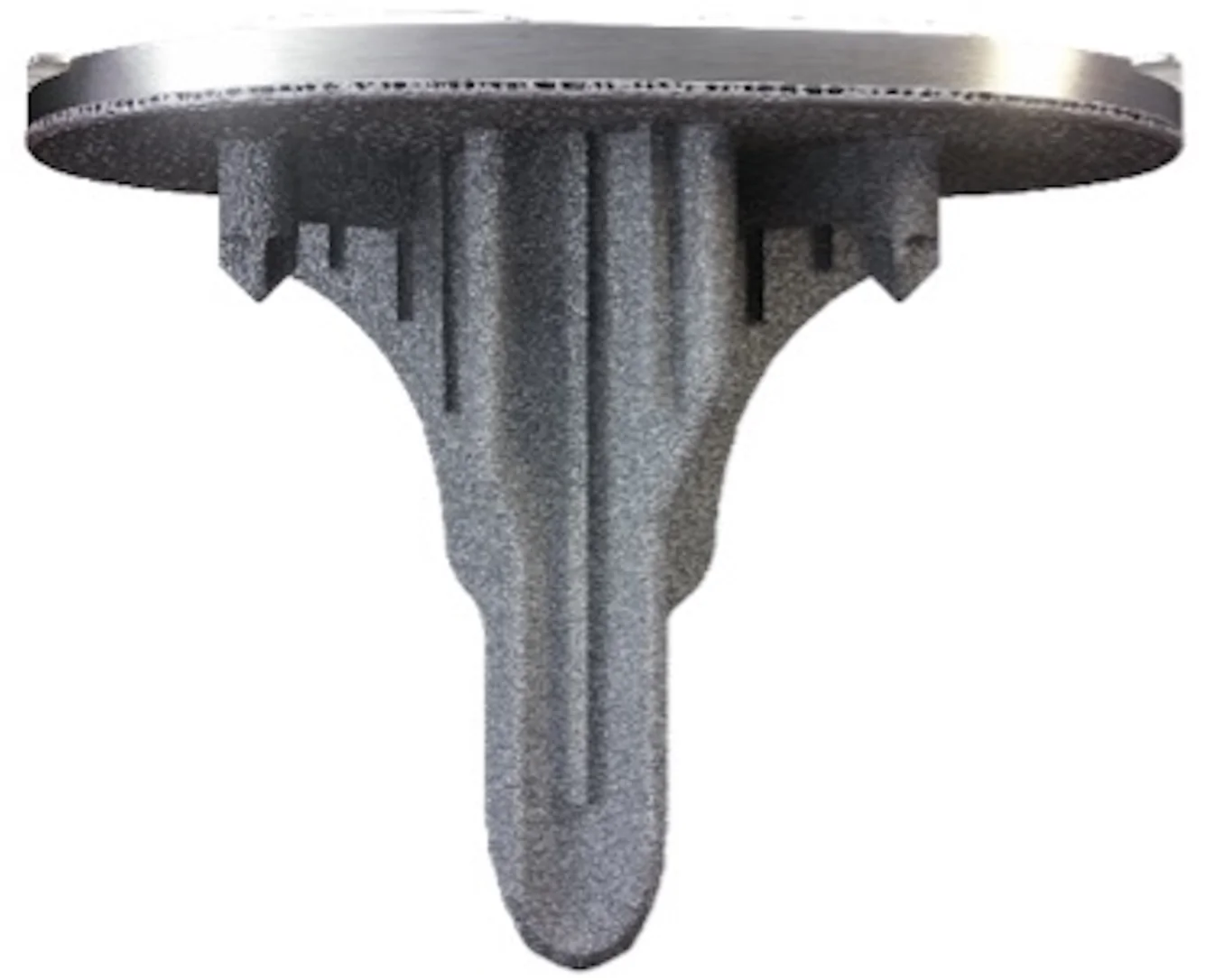
Anterior view of the studied anatomic, porous tibial baseplate.

**Figure 2:**
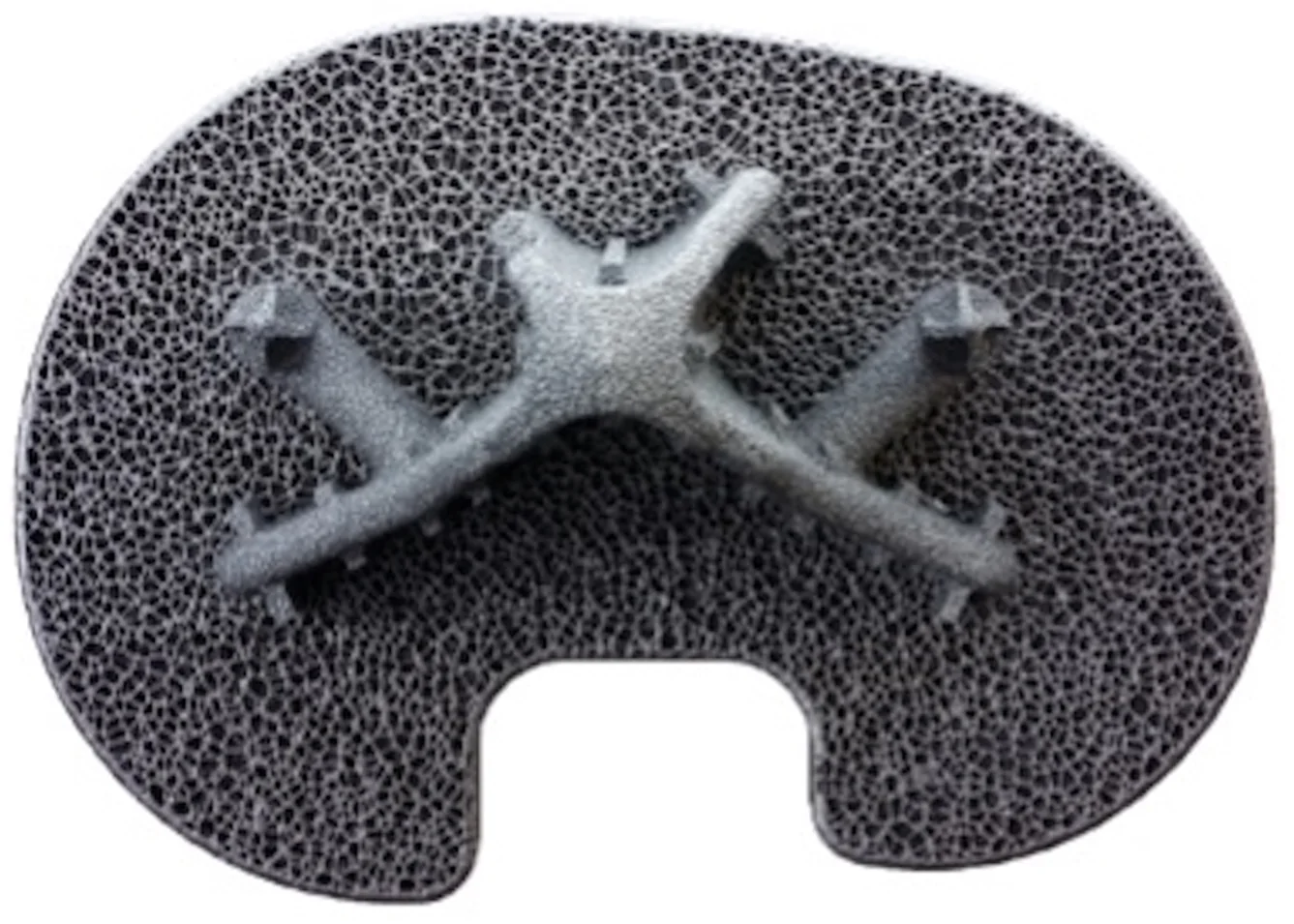
Inferior view of Smith and Nephew porous tibial baseplate.

The proprietary cementless asymmetric anatomic tibial baseplate is produced through an additive manufacturing process and consists of a three-dimensional printed titanium-aluminum-vanadium alloy with 80% porosity (range: 228–633 μm). The baseplate features a 3° posterior slope and a medialized anatomic keel with an overall length of 50 mm (Figs. 1 and 2) [12,13].

All TKAs were performed using an anterior midline skin incision using either a medial parapatellar or midvastus surgical approach. Patients received either the cementless or cemented tibial implant depending on the selected construct. All femoral components were cemented in both groups. Patellar resurfacing was performed according to surgeon preference. Standard postoperative anteroposterior and lateral radiographs were obtained. Patients were allowed immediate weight-bearing as tolerated, and physical therapy was initiated prior to discharge. Outpatient total knee arthroplasty continued postoperatively until maximal improvement in range of motion and strength was achieved, as determined by the treating surgeon and therapist.

Operative variables including surgical approach, use of robotic assistance, and patellar resurfacing were not standardized and were performed according to surgeon preference and routine clinical practice patterns at each participating institution. The choice of tibial fixation was determined intraoperatively by the treating surgeon and was not randomized. Selection of cementless fixation was based on surgeon assessment of factors including bone quality, patient age, BMI, and the anticipated ability to achieve durable biologic fixation, whereas cemented fixation was utilized when deemed more appropriate based on these considerations.

Follow-up visits occurred at approximately 2 weeks, 6 weeks, 6 months, 1 year, and annually thereafter, with some variation due to patient compliance. At each visit, clinical and radiographic evaluations were performed by orthopaedic surgeons to assess component loosening, radiolucency, fracture, subsidence, or reactive changes. Two orthopaedic surgeon reviewers were blinded to patient-reported outcomes and revision status at the time of radiographic review. Because fixation characteristics may be apparent radiographically, complete blinding to tibial fixation method could not be guaranteed.

The primary outcome was all-cause revision-free survivorship among the entire patient cohort. Aseptic tibial loosening-free survivorship was evaluated as a key secondary survivorship outcome given the low number of aseptic loosening events. Additional secondary outcomes included revision indication, manipulation under anesthesia (MUA), radiographic stability, Knee Injury and Osteoarthritis Outcome for Joint Replacement (KOOS-JR) scores, KOOS-JR change, and the proportion of patients achieving KOOS-JR Patient Acceptable Symptom State (PASS) and minimum clinically important difference (MCID) thresholds.

Functional outcomes were measured using the KOOS-JR both preoperatively and at 1 year postoperatively. KOOS-JR scores, which range from 0 to 100 (with 100 indicating perfect function), incorporate domains of pain, stiffness, function, and activities of daily living [14]. The proportion of patients achieving the PASS and MCID thresholds were compared between groups [15,16]. KOOS-JR change was calculated among patients with paired preoperative and 1-year postoperative scores. A multivariable linear regression model was used to evaluate the association between fixation group and KOOS-JR change while adjusting for baseline KOOS-JR, age, BMI, smoking status, and diabetes status. Effect estimates were reported as beta coefficients with 95% confidence intervals (CIs). Missing patient-reported outcome measures (PROM) data were handled using complete-case analysis; a sensitivity analysis compared baseline characteristics between patients with and without paired KOOS-JR data to assess potential missing-data bias. Given that the sensitivity analysis identified systematic differences between patients with and without paired scores, multiple imputation would represent a more robust approach to missing data and is a priority for future analyses.

Kaplan–Meier analysis was used to estimate implant survivorship, with all-cause revision as the failure event. Differences in survivorship between groups were assessed using the log-rank test. To account for unequal follow-up duration between cohorts, unadjusted Cox proportional hazards regression was performed with fixation group as the primary predictor. Due to the low number of revision events, which precluded adequate events-per-variable for reliable estimation, adjusted Cox regression was not performed. This analysis was repeated in a sensitivity cohort restricted to patients with at least 1 year of follow-up.

One-to-one propensity score matching without replacement was performed using nearest-neighbor matching with a caliper width of 0.2 standard deviations of the logit of the propensity score, using available baseline patient demographic and clinical variables with adequate overlap between groups. Due to the deidentified multicenter structure of the retrospective dataset, surgeon- and center-level identifiers were not consistently available for inclusion in the propensity score model. Surgical year also lacked sufficient overlap between cohorts because the cemented comparator cohort was primarily historical, whereas the cementless cohort included more contemporary cases; these variables therefore represent potential sources of residual confounding that could not be fully addressed through matching. Femoral fixation method was not included in the propensity score model because all femoral components were cemented in both cohorts. Covariate balance was assessed using standardized mean differences before and after matching, with values <0.1 indicating adequate balance.

No formal a priori power analysis was performed because this was a retrospective study of all available eligible cases. Given the low number of all-cause revision events and only 1 aseptic tibial loosening event in each cohort, the study was not powered to detect small or moderate differences in survivorship or to establish equivalence between fixation groups. Therefore, survivorship comparisons were interpreted as exploratory and hypothesis-generating.

Continuous variables were summarized as means with standard deviations, while categorical variables were reported as frequencies and percentages. Student’s *t*-tests were used for comparison of continuous variables, and chi-square or Fisher’s exact tests were used for categorical variables. All statistical analyses were performed in R version 4.4.0 (RStudio, Boston, MA). A *P* value <.05 was considered statistically significant.

## Results

A total of 394 patients were included in this study, with 183 in the cementless group and 211 in the cemented group (Table 1). The mean follow-up duration was 1.4 ± 1.1 years in the cementless group and 2.7 ± 1.9 years in the cemented group. Baseline characteristics were similar between groups, with the exception that the cementless group had significantly fewer smokers (*P* < .001).

**Table 1:** Patient demographics.

Variable	Cementless (n = 183)	Cemented (n = 211)	*P* value	SMD
Age, years	68.0 ± 8.3	68.2 ± 9.1	.80	0.026
Sex, *n* (%)			.97	0.008
Male	62 (33.9%)	70 (33.2%)		
Female	121 (66.1%)	141 (66.8%)		
BMI, kg/m^2^	33.4 ± 6.8	33.3 ± 6.6	.91	0.011
Laterality, *n* (%)			.48	0.190
Left	88 (48.1%)	115 (54.5%)		
Right	93 (50.8%)	96 (45.5%)		
Bilateral	2 (1.1%)	0 (0%)		
Diabetes, *n* (%)	35 (19.1%)	41 (19.4%)	1.00	0.008
Current smoker, *n* (%)	6 (3.3%)	73 (34.6%)	<.001	0.872
Mean follow-up, years	1.4 ± 1.1	2.7 ± 1.9	<.001	0.838
Radiographic follow-up, days	423.4 ± 186.5	988.8 ± 819.9	<.001	0.951

Values are presented as mean ± standard deviation or *n* (%). Standardized mean differences are reported for between-group balance, with SMD >0.10 considered potentially meaningful imbalance.

SMD, standardized mean difference.

**Figure 3:**
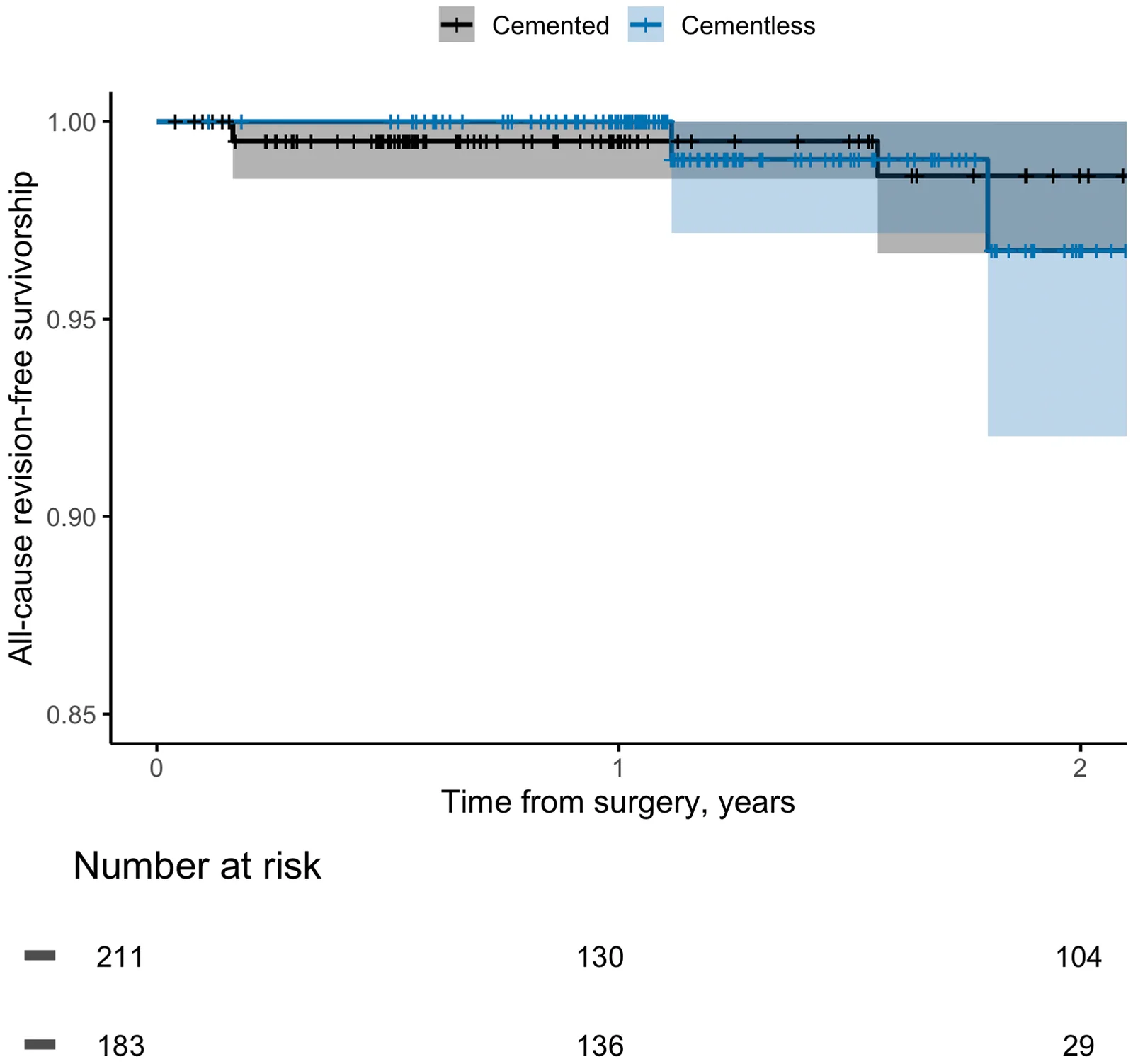
Kaplan–Meier analysis of all-cause revision-free survivorship following primary total knee arthroplasty for cementless vs cemented tibial baseplates. Shaded areas represent 95% confidence intervals. Differences in survivorship between fixation methods are shown up to 2 years postoperatively (log-rank *P* = .93), with numbers at risk displayed below the x-axis.

The revision rate during the follow-up period was 1.6% (n = 3) in the cementless group (Table 2). Overall revision-free survivorship was 98.8% at 1 year and 96.2% at 2 years. In the cemented group, the revision rate was 2.8% (n = 6), with revision-free survivorship of 99.5% at 1 year and 96.0% at 2 years (Table 3, Fig. 3). At 2 years, the number at risk was substantially lower in the cementless cohort than in the cemented cohort, reflecting the shorter follow-up duration of the cementless group; 2-year survivorship estimates for this cohort should therefore be interpreted with caution.

**Table 2:** Early clinical outcomes.

Outcome	Cementless (n = 183)	Cemented (n = 211)	*P*-value
All-cause revision, *n* (%)	3 (1.6%)	6 (2.8%)	.53
Aseptic loosening revision, *n* (%)	1 (0.5%)	1 (0.5%)	1.00
Time to revision, days	419.3 ± 214.3	798.0 ± 861.2	—
Manipulation under anesthesia, *n* (%)	10 (5.5%)	12 (5.7%)	.94

Values reported as mean ± standard deviation unless otherwise indicated. Categorical variables were compared with Chi-square or Fisher’s exact tests. Time to revision reported descriptively due to low event counts.

**Table 3:** Kaplan–Meier all-cause revision-free survivorship.

Time point	Cementless survivorship (95% CI)	n at risk	Cemented survivorship (95% CI)	n at risk
1 year	98.8% (97.1–100.0)	136	99.5% (98.5–100.0)	130
2 years	96.2% (91.2–100.0)	29	96.0% (92.6–99.6)	104

There were no significant differences between groups in all-cause revision or revision for aseptic loosening. Reported reasons for revision included isolated cases of anterior knee pain, aseptic tibial baseplate loosening, and periprosthetic joint infection (Supplementary Table 2). Mean time to revision was 419.3 ± 214.3 days in the cementless cohort and 798 ± 861.2 days in the cemented cohort. MUA was required in 10 patients (5.5%) in the cementless group and 12 patients (5.7%) in the cemented group (*P* = .94).

The mean radiographic follow-up period was 423.4 ± 186.5 days for the cementless group and 988.8 ± 819.9 days for the cemented group. At final follow-up, independent radiographic review demonstrated no evidence of component loosening, malalignment, or fracture in the remaining unrevised cases. Both reviewers identified the same 2 cases of aseptic tibial loosening, 1 in each cohort, with all other tibial components classified as radiographically stable relative to prior imaging. Interobserver agreement for tibial loosening classification was 100%, and both loosening cases were confirmed at the time of revision.

In unadjusted Cox proportional hazards regression (not controlling for patient-level covariates due to the low number of revision events), cementless fixation was not significantly associated with all-cause revision in the overall cohort (hazard ratio 3.27, 95% CI 0.52–20.53; *P* = .207) or in the ≥1-year follow-up sensitivity cohort (hazard ratio 7.36, 95% CI 0.90–60.31; *P* = .063). Both estimates were imprecise due to the low number of revision events and should be interpreted accordingly (Supplementary Table 1).

For postoperative KOOS-JR, PASS, and other 1-year cross-sectional comparisons, all patients with an available 1-year KOOS-JR assessment were included (cementless n = 131; cemented n = 119). Analyses of KOOS-JR change and MCID achievement were restricted to patients with paired preoperative and 1-year postoperative KOOS-JR data (cementless n = 69; cemented n = 118), as calculation of change scores required both assessments (Table 4). Preoperatively, KOOS-JR scores were 42.8 ± 15.8 in the cementless group and 46.6 ± 13.2 in the cemented group (*P* = .10). At 1 year postoperatively, KOOS-JR scores were 74.0 ± 16.5 in the cementless group and 69.7 ± 13.5 in the cemented group (*P* = .07). There was no significant difference between groups in the proportion of patients achieving the KOOS-JR PASS or the MCID (*P* = .12 and *P* = .40, respectively).

**Table 4:** Patient-reported outcomes.

Outcome	Cementless	Cemented	*P* value
KOOS JR preoperative	42.8 ± 15.8 (n = 69)	46.6 ± 13.2 (n = 118)	.10
KOOS JR postoperative (1 year)	74.0 ± 16.5 (n = 131)	69.7 ± 13.5 (n = 119)	.07
KOOS JR change	31.3 ± 23.2	23.1 ± 18.9	.015
KOOS JR PASS (≥71), n/N (%)	74/131 (56.5%)	52/119 (43.7%)	.12
KOOS JR MCID achieved, n/N (%)	61/69 (88.4%)	99/118 (83.9%)	.40

Postoperative KOOS-JR and PASS analyses included all patients with available 1-year postoperative KOOS-JR data. KOOS-JR change and MCID analyses were limited to patients with paired preoperative and 1-year postoperative KOOS-JR assessments. Change indicates the difference between preoperative and postoperative KOOS-JR scores.

In unadjusted analysis, mean KOOS-JR improvement was greater in the cementless cohort (31.3 ± 23.2 points) than in the cemented cohort (23.1 ± 18.9 points; *P* = .015). However, the cementless group had lower mean preoperative KOOS-JR scores, and this baseline difference is known to inflate apparent improvement through regression to the mean. After adjustment for baseline KOOS-JR, age, BMI, smoking status, and diabetes status in multivariable linear regression, cementless fixation was not significantly associated with KOOS-JR change compared with cemented fixation (β = 3.46 points; 95% CI, −1.31 to 8.24; *P* = .154). Baseline KOOS-JR was strongly and inversely associated with KOOS-JR change (β = −1.06; 95% CI, −1.21 to −0.91; *P* < .001), confirming that patients with lower preoperative scores improved more regardless of fixation type, consistent with the tendency for lower-functioning patients to show disproportionately larger gains irrespective of the treatment received. The sensitivity analysis for missing PROM data identified significant differences in age, BMI, smoking status, and fixation group between patients with and without paired KOOS-JR scores, indicating that outcome data were not missing at random and suggesting potential response bias; PROM findings should, therefore, be interpreted with caution.

After propensity score matching to control for baseline differences, 143 patients remained in each group (Supplementary Table 3). In the matched cohort, rates of all-cause revision and 2-year survivorship remained low in both the cementless and cemented groups. Because this analysis did not account for surgeon, center, or surgical era, findings from the matched cohort should be interpreted cautiously (Supplementary Table 4).

## Discussion

Cementless TKA has experienced a resurgence in recent years [1,2]. Modern cementless tibial components, particularly those using keel-and-peg configurations, have demonstrated promising survivorship with low rates of aseptic loosening [7,9]. The present study evaluated the early performance of a new cementless tibial baseplate compared with a modern cemented counterpart from the same manufacturer.

Aseptic loosening is one of the most common causes of TKA failure beyond 2 years [17]. When comparing fixation techniques, several studies have reported higher early rates of aseptic loosening among cementless cohorts [5,10]. Tibial aseptic loosening occurs more frequently than femoral loosening, likely due to differences in load distribution, implant micromotion, and biomechanics of the bone–implant interface [18,19]. Because the tibial component endures greater compressive and shear forces, it is inherently more susceptible to early instability, emphasizing the need for implant designs that optimize load transfer and biologic fixation. As patient expectations increase and younger, more active individuals undergo TKA, addressing tibial aseptic loosening remains a critical challenge for short- and long-term implant survivorship.

In the present study, aseptic loosening and all-cause revision rates did not differ significantly between cementless and cemented tibial components. Notably, mean time to revision was shorter in the cementless group, and the cementless cohort had a significantly shorter mean follow-up duration overall. This disparity limits the ability to fully evaluate differences in long-term survivorship between groups. Although unadjusted Cox regression did not demonstrate a statistically significant difference in all-cause revision, the hazard ratio estimates consistently favored the cemented cohort and were associated with wide CIs because of the small number of revision events. Consequently, these findings should not be interpreted as evidence of equivalent survivorship between fixation strategies.

The porous anatomic tibial baseplate evaluated in this study was designed to maximize proximal tibial coverage and distribute mechanical stress more evenly [20]. Two antirotation pegs provide rotational stability during early postoperative loading, and a medialized anatomic keel increases the surface area available for osseointegration [21]. The baseplate’s three-dimensional printed titanium alloy structure offers a consistent and highly porous architecture intended to facilitate rapid bone ingrowth [22,23]. Prior preclinical studies have demonstrated robust bone ingrowth and mechanical fixation of highly porous additively manufactured titanium implant surfaces in load-bearing models [23,24].

Successful osseointegration requires sufficient initial mechanical stability to enable biologic fixation. According to Perren’s strain theory, excessive interfacial micromotion favors fibrous encapsulation over bone formation, potentially leading to implant failure [[25], [26], [27]]. Although mature osseointegration typically occurs by 9-12 months postoperatively, ex vivo culture studies have demonstrated new bone formation on implant surfaces as early as 7 weeks [28], and animal models have shown early bone on-growth at 4 and 12 weeks with continued improvements over time [29]. The porous architecture of the tibial baseplate in the present study is intended to provide a biologically favorable environment during early osseointegration, although this study was not designed to determine whether these design features reduce long-term micromotion-related failure.

In our study, the cementless tibial baseplate demonstrated an aseptic loosening rate of 0.5% (n = 1), corresponding to a 99.5% aseptic revision-free survivorship at a mean follow-up of 1.4 years. This is consistent with prior reports of similar keel-and-peg cementless designs, which have demonstrated aseptic loosening rates ranging from 1.0% at 2-year follow-up [9] to 2.7% in other designs [10]. The cemented tibial baseplate used for comparison demonstrated a similarly low observed aseptic loosening revision rate (0.5%); however, the small number of events and unequal follow-up duration limit definitive comparisons between fixation strategies. Notably, a recent cohort comparison reported substantially higher early aseptic loosening with a different cementless tibial baseplate design (7% vs 0.5%) at 12 months [11], underscoring that outcomes may be implant design-specific rather than class-specific.

These findings align with previous studies showing favorable early survivorship of cemented tibial components, including case series reporting no aseptic tibial loosening at 3.5-year follow-up [30] and large registry-based reviews documenting aseptic revision rates of 0.7% over 4 years [31].

Among the revision cases observed in this study, 1 patient experienced anterior knee pain attributed to the absence of patellar resurfacing, 1 patient with a cancer history developed a periprosthetic joint infection, and 1 patient with postoperative stiffness underwent MUA followed by revision TKA for aseptic tibial loosening. The patient in the cementless group who required revision at 322 days had a BMI of 39.1, compared to 451 days and a BMI of 29.3 in the cemented group. The relationship between BMI and osseointegration is complex: while increased mechanical loading has been proposed to promote bone ingrowth in some contexts [[32], [33], [34]], excessive loading may also compromise early fixation in cementless constructs, and no firm conclusions can be drawn from a single case. Cemented implants may also demonstrate longer time to failure because cement provides immediate mechanical stability [35], whereas cementless implants require several months to achieve full biologic fixation, a period during which insufficient early stability can increase the risk of loosening.

Despite the low revision rate, PROMs were favorable overall. KOOS-JR scores averaged 74.0 ± 16.5 in the cementless group, which was not significantly different from the cemented cohort (69.7 ± 13.5; *P* = .07). In unadjusted analysis, the cementless cohort demonstrated greater KOOS-JR improvement; however, this difference was no longer statistically significant after multivariable adjustment for baseline KOOS-JR and patient characteristics, suggesting that the apparent unadjusted advantage reflects baseline score imbalance rather than a true treatment effect, as patients with lower preoperative function characteristically demonstrate larger gains independent of the intervention received. Additionally, nonrandom patterns of missing paired PROM data may have introduced response bias, further limited the interpretability of these findings.

This study has several limitations that should be considered when interpreting findings. First, the retrospective design and nonrandomized selection of fixation method introduce the potential for selection bias, as tibial fixation was determined intraoperatively by the treating surgeon based on assessment of bone quality, patient age, and BMI. Second, the cemented comparator cohort was historical and differed from the cementless cohort with respect to surgeon, institution, and surgical era; because these variables could not be consistently captured within the deidentified multicenter dataset, residual confounding remains possible and cannot be fully addressed through propensity score matching. Third, follow-up duration was unequal between cohorts, which may independently influence PROMs, radiographic findings, and aseptic loosening detection, as these are time-dependent processes; observed differences may therefore partially reflect variation in observation time rather than fixation strategy alone. Fourth, the low incidence of revision, aseptic loosening events, and MUA severely limits statistical power to detect clinically meaningful differences between groups, and the resulting wide CIs preclude conclusions regarding equivalence. Fifth, several operative variables, including surgical approach, patellar resurfacing, and use of robotic assistance, were not standardized and may independently influence outcomes, introducing additional residual confounding. Sixth, non-random loss of paired KOOS-JR data, as identified in the sensitivity analysis, may have introduced response bias that complete-case analysis cannot fully address. Finally, the findings should not be interpreted as evidence of equivalence between fixation strategies; selection bias, residual confounding, and chance remain plausible explanations for the low early revision rates observed in both cohorts.

Despite these limitations, the present study contributes meaningful early observational evidence regarding a novel cementless tibial baseplate design, providing insight into short-term survivorship and functional recovery. Future prospective studies with longer and more uniform follow-up standardized operative protocols, and contemporary cemented controls are warranted to evaluate long-term outcomes of cementless vs cemented tibial fixation.

## Conclusions

In this multicenter retrospective cohort, a modern anatomic cementless tibial baseplate demonstrated low early rates of all-cause revision and aseptic tibial loosening within the available follow-up period. Interpretation is limited by the retrospective design, historical cemented comparator cohort, unequal follow-up duration, low event counts, and residual confounding. These findings should be considered preliminary device-specific observational data, and longer-term prospective studies with contemporary controls are needed before definitive conclusions regarding comparative survivorship can be drawn.

## CRediT authorship contribution statement

**Alexander Burbelo:** Writing – review & editing, Writing – original draft, Investigation, Formal analysis, Data curation, Conceptualization. **Rex Lutz:** Writing – review & editing, Writing – original draft, Methodology, Investigation, Formal analysis, Data curation, Conceptualization. **Steven Fischer:** Writing – review & editing, Writing – original draft, Investigation, Data curation, Conceptualization. **Harrison Patrizio:** Writing – review & editing, Writing – original draft, Investigation, Data curation, Conceptualization. **Marcus Voigt:** Writing – review & editing, Writing – original draft, Investigation. **Liam Cleary:** Writing – review & editing, Writing – original draft, Investigation, Data curation. **Trace Clark:** Writing – review & editing, Writing – original draft, Investigation. **Arjun Saxena:** Writing – review & editing, Writing – original draft, Supervision, Methodology, Investigation, Data curation, Conceptualization. **Matthew Bullock:** Writing – review & editing, Writing – original draft, Project administration, Methodology, Investigation, Data curation, Conceptualization. **Alvin Ong:** Writing – review & editing, Writing – original draft, Project administration, Methodology, Investigation, Formal analysis, Data curation, Conceptualization.

## Conflict of interest

Alvin Ong receives honoraria for speakers bureau activities for Stryker, Smith and Nephew; is a paid consultant for Stryker, Smith and Nephew; receives research support from Smith and Nephew; receives royalties, financial or material support from Smith and Nephew; Arjun Saxena holds stock or stock options in Parvizi Surgical Innovations; receives research support from United Orthopaedics; serves on the JOA editorial or governing board, JBJS editorial or governing board, Journal of Surgical Advances editorial or governing board, JAAOS editorial or governing board; and also serves as an AAOS Board or committee member, AAHKS Board or committee member, ABOS Board or committee member, EOA Board or committee member, NJOS Board or committee member, Pennsylvania Orthopaedic Society Board or committee member. Matthew Bullock receives honoraria for speakers bureau activities for Smith and Nephew; is a paid consultant for Smith and Nephew; and serves as a board member for the West Virginia Orthopaedic Education Committee, JOA Editorial board, Arthroplasty today editorial board—He has recused himself from the peer review process; Rex Luztz serves as a Board member or committee appointments for Chair AAOS Resident Assembly Executive Committee. All other authors declare no potential conflicts of interest.

For full disclosure statements refer to https://doi.org/10.1016/j.artd.2026.102117.

## Figures and Tables

**Supplementary Table 1:** Cox proportional hazards models for all-cause revision

Analysis cohort	Cemented, n	Cementless, n	Total revision events	Hazard ratio for cementless vs cemented	95% CI	*P* value
Overall cohort	211	183	9	3.27	0.52–20.53	.207
≥1-year follow-up sensitivity cohort	131	140	8	7.36	0.90–60.31	.063

Cox proportional hazards models were unadjusted and included fixation group as the sole predictor. Hazard ratios compare cementless fixation with cemented fixation as the reference group. Models were intentionally not adjusted for additional covariates because of the low number of all-cause revision events, which would increase the risk of model overfitting.

**Supplementary Table 2:** Indications for revision

Indication	Cementless (n = 3)	Cemented (n = 6)
Aseptic loosening	1	1
Instability	0	4
Periprosthetic joint infection	1	0
Anterior knee pain	1	0
Traumatic wound dehiscence	0	1

**Supplementary Table 3:** Baseline characteristics after 1:1 propensity score matching

Variable	Cementless (n = 143)	Cemented (n = 143)	SMD
Age, years	68.4 ± 8.1	69.8 ± 8.8	0.17
Male sex, n (%)	37 (25.9%)	35 (24.5%)	0.03
BMI, kg/m^2^	33.3 ± 6.8	32.9 ± 6.8	0.06
Diabetes, *n* (%)	25 (17.5%)	29 (20.3%)	0.07
Current smoker, *n* (%)	6 (4.2%)	6 (4.2%)	<0.001

SMD, standardized mean difference.

**Supplementary Table 4:** Matched cohort (1:1, n = 143 per group)

Outcome	Cementless (n = 143)	Cemented (n = 143)	*P* value
All-cause revision, *n* (%)	2 (1.4%)	2 (1.4%)	1.00^a^
2-year survivorship, % (95% CI)	95.2% (87.8–100)	98.7% (96.1–100)	.30^b^

a Fisher’s exact test.

b Log-rank test.

## Appendix A Supplementary data

Conflict of Interest Statement for Ong

Conflict of Interest Statement for Patrizio

Conflict of Interest Statement for Cleary

Conflict of Interest Statement for Bullock

Conflict of Interest Statement for Voigt

Conflict of Interest Statement for Lutz

Conflict of Interest Statement for Fischer

Conflict of Interest Statement for Clark

Conflict of Interest Statement for Saxena

Conflict of Interest Statement for Burbelo

## References

[1] Mosher Z.A., Bolognesi M.P., Malkani A.L., Meneghini R.M., Oni J.K., Fricka K.B. (2024). Cementless total knee arthroplasty: a Resurgence-Who, when, where, and how?. J Arthroplasty.

[2] Garvin K.L., Harrast J.J., Nelson C.L., Jacobs J.J., Martin D.F. (2025). Primary total hip arthroplasty performed in 2022 to 2023 by candidates for the American board of orthopaedic surgery board certification and recertification: fixation and patient Age. J Arthroplasty.

[3] Puri S., Alpaugh K., Chiu Y.-F., Ast M.P., Jerabek S., Westrich G. (2024). Cementless versus cemented total knee arthroplasty of the same design: shorter operative times and minimal differences in early outcomes. HSS J.

[4] Miller A.J., Stimac J.D., Smith L.S., Feher A.W., Yakkanti M.R., Malkani A.L. (2018). Results of cemented vs cementless primary total knee arthroplasty using the same implant design. J Arthroplasty.

[5] Forlenza E.M., Serino J., Terhune E.B., Weintraub M.T., Nam D., Della Valle C.J. (2023). Cementless total knee arthroplasty is associated with early aseptic loosening in a large national database. J Arthroplasty.

[6] Chen F., Chang R.N., Prentice H.A., Fasig B.H., Paxton E.W., Hug K.T. (2025). What is the survivorship of TKA with a twin-peg or spikes-and-keel cementless implant compared with cemented? A registry-based cohort Study. Clin Orthop Relat Res.

[7] Helvie P.F., Deckard E.R., Meneghini R.M. (2023). Cementless total knee arthroplasty over the past decade: excellent survivorship in contemporary designs. J Arthroplasty.

[8] (2024). https://www.aahks.org/wp-content/uploads/2024/09/2024-Hip-and-Knee.pdf.

[9] Yazdi H., Choo K.J., Restrepo C., Hammad M., Sherman M., Parvizi J. (2020). Short-term results of triathlon cementless versus cemented primary total knee arthroplasty. Knee.

[10] Gibian J.T., Zuke W.A., Hood H., Blum E., Nunley R.M., Barrack R.L. (2025). Early aseptic tibial loosening is a concern with a modern two-peg cementless total knee arthroplasty design. J Arthroplasty.

[11] Andronic O., Yang Y.H., Pabbruwe M., Jones C.W., Yates P.J. (2025). Early aseptic loosening and inferior patient-reported outcomes of a cementless tibial baseplate in a modern total knee arthroplasty design. Bone Joint J.

[12] Journey II total knee arthroplasty. https://www.smith-nephew.com/en-us/health-care-professionals/products/orthopaedics/journey-ii-tka.

[13] LEGION CONCELOC cementless total knee System. https://www.smith-nephew.com/en-us/health-care-professionals/products/orthopaedics/legion-conceloc.

[14] Roos E.M., Lohmander L.S. (2003). The Knee injury and Osteoarthritis Outcome Score (KOOS): from joint injury to osteoarthritis. Health Qual Life Outcomes.

[15] Dekhne M.S., Fontana M.A., Pandey S., Driscoll D.A., Lyman S., McLawhorn A.S. (2024). Defining patient-relevant thresholds and change scores for the HOOS JR and KOOS JR anchored on the patient-acceptable symptom State question. Clin Orthop Relat Res.

[16] Emara A.K., Pasqualini I., Jin Y., Klika A.K., Orr M.N., Rullán P.J. (2024). Diagnosis-Specific thresholds of the minimal clinically important difference and patient acceptable symptom State for KOOS after total knee arthroplasty. J Bone Joint Surg Am.

[17] Sharkey P.F., Lichstein P.M., Shen C., Tokarski A.T., Parvizi J. (2014). Why are total knee arthroplasties failing today--has anything changed after 10 years?. J Arthroplasty.

[18] Conditt M.A., Parsley B.S., Alexander J.W., Doherty S.D., Noble P.C. (2004). The optimal strategy for stable tibial fixation in revision total knee arthroplasty. J Arthroplasty.

[19] Baek J.-H., Lee S.C., Ryu S., Ahn H.S., Nam C.H. (2022). Early aseptic loosening of primary total knee arthroplasty in patients with osteonecrosis of the knee: a case series. Clin Case Rep.

[20] Ho J.P.Y., Park S.Y., Nam H.S., Cho J.H., Lee Y.S. (2023). The use of an anatomic tibial baseplate in total knee arthroplasty optimizes coverage without compromising rotation: a propensity-matched evaluation. Knee.

[21] Bhimji S., Meneghini R.M. (2014). Micromotion of cementless tibial baseplates: keels with adjuvant pegs offer more stability than pegs alone. J Arthroplasty.

[22] Phuoc H.D., Hoang P.N., Yang S., Fraser D., Nguyen V.T. (2023). Osseointegrability of 3D-printed porous titanium alloy implant on tibial shaft bone defect in rabbit model. PLoS One.

[23] Chen Y.-S., Wu P.-K., Tsai W.-C., Lin C.-L. (2025). Enhancing bone ingrowth and mechanical bond strength at the titanium alloy 3D-printed implant-bone interface through advanced lattice designs and growth factor integration. IJB.

[24] LEGION CONCELOC Cementless total Knee System tested for biologic fixation and osseointegration. https://snnvisionaire.com/media/bcihpqjx/28725-v1-legion-tks-with-conceloc-design-rationale-1021.pdf.

[25] Glatt V., Evans C.H., Tetsworth K. (2021). Reverse dynamisation: a modern perspective on Stephan Perren’s strain theory. Eur Cell Mater.

[26] Perren S.M. (1979). Physical and biological aspects of fracture healing with special reference to internal fixation. Clin Orthop Relat Res.

[27] Wazen R.M., Currey J.A., Guo H., Brunski J.B., Helms J.A., Nanci A. (2013). Micromotion-induced strain fields influence early stages of repair at bone-implant interfaces. Acta Biomater.

[28] Dua R., Jones H., Noble P.C. (2021). Evaluation of bone formation on orthopedic implant surfaces using an ex-vivo bone bioreactor system. Sci Rep.

[29] Walsh W.R., Pelletier M.H., Bertollo N., Lovric V., Wang T., Morberg P. (2020). Bone ongrowth and mechanical fixation of implants in cortical and cancellous bone. J Orthop Surg Res.

[30] Christen B., Neukamp M., Aghayev E. (2014). Consecutive series of 226 journey bicruciate substituting total knee replacements: early complication and revision rates. BMC Musculoskelet Disord.

[31] Harris A.I., Christen B., Malcorps J.J., O’Grady C.P., Kopjar B., Sensiba P.R. (2019). Midterm performance of a guided-motion bicruciate-stabilized total knee System: results from the international Study of over 2000 consecutive primary total knee arthroplasties. J Arthroplasty.

[32] Chavarri-Prado D., Brizuela-Velasco A., Álvarez-Arenal Á., Dieguez-Pereira M., Pérez-Pevida E., Viteri-Agustín I. (2020). The bone buttress theory: the effect of the mechanical loading of bone on the osseointegration of dental implants. Biology (Basel).

[33] Meisterhans M., Dimitriou D., Fasser M., Hoch A., Jud L., Zingg P.O. (2024). Influence of offset on osseointegration in cementless total hip arthroplasty: a finite element study. J Orthop Research.

[34] Sinicrope B.J., Feher A.W., Bhimani S.J., Smith L.S., Harwin S.F., Yakkanti M.R. (2019). Increased survivorship of Cementless versus cemented TKA in the morbidly Obese. A minimum 5-Year Follow-Up. J Arthroplasty.

[35] Choi B.S., Jung M., Cho B.W., Chung J.Y., Ha J.K., Han H.-S. (2026). Patient selection and management for successful cementless total knee arthroplasty. Knee Surg Relat Res.

