# Supplementary material for: Early Survivorship Analysis of a Modern Anatomic Cementless Tibial Baseplate: A Multicenter Retrospective Study
Source: Arthroplast Today. 2026 Aug 12;40:102117. doi: 10.1016/j.artd.2026.102117 (PMC13495097; doi:10.1016/j.artd.2026.102117)
Supplement: Supplementary file 10 [file mmc9.docx]

# INDIVIDUAL CONFLICT OF INTEREST STATEMENT

***American Association of Hip and Knee Surgeons***

(Adopted from the American Academy of Orthopaedic Surgeons disclosure statement)

The following form **must be filled out completely and submitted by each author (example, 6 authors, 6 forms).**

**All items require a response. If there is no relevant disclosure for a given item, enter "*None*.”**

**Manuscript Title**: Early Survivorship Analysis Of a Modern Anatomic Cementless Tibial Baseplate: A Multicenter Retrospective Study

1.	Royalties from a company or supplier (The following conflicts were disclosed)

None

2.	Speakers bureau/paid presentations for a company or supplier (The following conflicts were disclosed)

None

3A.	Paid employee for a company or supplier (The following conflicts were disclosed)

None

3B.	Paid consultant for a company or supplier (The following conflicts were disclosed)

Corin

3C.	Unpaid consultants for a company or supplier (The following conflicts were disclosed)

None

4.	Stock or stock options in a company or supplier (The following conflicts were disclosed)

Parvizi Surgical Innovations

5.	Research support from a company or supplier as a Principal Investigator (The following conflicts were disclosed)

United Orthopaedics

6.	Other financial or material support from a company or supplier (The following conflicts were disclosed)

None

7.	Royalties, financial or material support from publishers (The following conflicts were disclosed)

None

8.	Medical/Orthopaedic publications editorial/governing board (The following conflicts were disclosed)

JOA editorial or governing board, JBJS editorial or governing board, Journal of Surgical Advances editorial or governing board, JAAOS editorial or governing board

9.	Board member/committee appointments for a society (The following conflicts were disclosed)

AAOS Board or committee member, AAHKS Board or committee member, ABOS Board or committee member, EOA Board or committee member, NJOS Board or committee member, Pennsylvania Orthopaedic Society Board or committee member

**Each author must sign AND print or type his/her name, date and submit a separate form**

In addition, one BLINDED Conflict of Interest form (no author names used) should be submitted per manuscript with all author disclosures.

Arjun Saxena	Arjun Saxena	4.6.25

Author Name (Print or Type)	Author Signature	Date
